# Speech-Language Pathology Interventions in Pediatric Awake Brain Surgery: A Moroccan and Global Perspective

**DOI:** 10.7759/cureus.77609

**Published:** 2025-01-18

**Authors:** Nada Benabbou, Saber Boutayeb, Kawtar Chafik, Hicham Lafhal, Mohammed Elhassani

**Affiliations:** 1 Clinical Epidemiology and Medico-Surgical Science and Translational Oncology, Faculty of Medicine and Pharmacy, Mohammed V University, Rabat, MAR; 2 Oncology and Gynecologic Oncology, Faculty of Medicine, Mohammed V University, Rabat, MAR; 3 Health and Nutrition of Mother and Child, Faculty of Medicine and Pharmacy, Mohammed V University, Rabat, MAR; 4 Rehabilitation, Higher Institute of Nursing Professions and Health Techniques, Rabat, MAR; 5 Neurosurgery, Ibn Sina University Hospital Center, Rabat, MAR

**Keywords:** awake surgery, brain mapping, cognitive and linguistic assessment, pediatric, speech therapy

## Abstract

Introduction

Awake brain surgery (ABS) is a groundbreaking technique that not only enhances tumor resection but also preserves vital neurocognitive functions, particularly through advanced brain mapping. Despite its success in adults, ABS in pediatric patients remains significantly underexplored, especially concerning the role of speech-language pathology (SLP) in these procedures. This study addresses this gap by providing a thorough examination of SLP interventions in pediatric ABS across various university hospitals, including those in Morocco and internationally.

Methods

We utilized a web-based survey, translated into French, English, and Spanish, to capture the clinical practices of 10 pediatric ABS teams globally, including one team at Rabat University Hospital in Morocco. The survey included questionnaires tailored for neurosurgeons, speech therapists, and other professionals involved in ABS. Additionally, a detailed review of medical records for three pediatric patients who underwent ABS in Morocco between 2017 and 2022 was conducted. Data were analyzed using SPSS (IBM Corp., Armonk, NY, USA) for quantitative results and qualitative content analysis for case study evaluations.

Results

The study found a global consensus on the essential role of SLP in pediatric ABS, with 62.5% of neurosurgeons stressing the need for specialized SLP training. A significant correlation was observed between postoperative speech therapy and reduced neurolinguistic disorders (Pearson correlation=0.340, p=0.007). Challenges in assessing written language components were identified due to a lack of suitable tools, highlighting the need for improved resources. Additionally, most speech assessments during brain mapping were conducted in the patient's mother tongue (60.7%), and assessments were most commonly performed during all three stages of surgery (25%).

Conclusion

This study emphasizes the importance of speech therapy in pediatric ABS, highlighting the need for specialized tools and training. Findings suggest a positive impact of postoperative speech therapy on neurolinguistic outcomes, but further research with larger cohorts and long-term follow-up is needed to confirm these benefits.

## Introduction

Awake brain surgery (ABS) is now widely recognized as the gold standard in neurosurgical oncology, particularly for treating conditions like slow-growing infiltrating brain tumors, epilepsy, and arteriovenous malformations [1-6]. The primary objective of ABS is to maximize the removal of cancer while preserving essential functions such as motor, sensory, language, and cognitive abilities [7-10]. By keeping the patient awake during surgery, neurosurgeons can perform real-time anatomy-functional cortical and subcortical mapping, which aids in protecting critical brain regions, leading to better patient outcomes and preserving postoperative quality of life [11,12].

One of the key players in ABS, especially when language and cognitive functions are involved, is the speech-language pathologist (SLPs) [13,14]. SLPs are integral to the surgical process at every stage [15]. Preoperatively, they assess the patient’s language abilities to establish a baseline. During surgery, SLPs monitor language performance as the brain is stimulated to preserve eloquent brain regions. Postoperatively, they assess and treat any deficits that may arise. While the role of SLPs in ABS is well-documented in adult patients, there is a notable lack of research on their involvement in pediatric populations, even though children undergoing ABS have unique developmental challenges [1,16-17].

Pediatric patients present additional complexities compared to adults in ABS. Children’s brains exhibit high neuroplasticity, meaning they may recover and adapt differently from adults, especially regarding language acquisition and cognitive development [18]. Moreover, psychological factors are critical in determining whether a child can participate in ABS. While children are typically considered for awake surgery around the age of five, this decision is not based solely on age but also on the child’s emotional and cognitive maturity. Some children may struggle with anxiety or behavioral issues, making cooperation during surgery difficult, while others may be more receptive to the procedure [19].

A significant issue in pediatric ABS is the lack of age-appropriate assessment tools for language and cognitive function. Commonly used tools, such as the Boston Diagnostic Aphasia Examination (BDAE) and the Picture Naming Test (DO80), were initially developed for adults and are often adapted for pediatric use. However, these modifications may not fully account for developmental differences, highlighting the need for pediatric-specific tools.

In addition to the technical challenges, psychosocial aspects play a crucial role in pediatric ABS. Preoperative preparation, both psychological and emotional, is essential to the success of the procedure. Children need to understand the surgical process in a way appropriate for their age, often through visual aids, familiarization with the surgical environment, and support from parents and caregivers. The psychological readiness of the child, as well as that of the caregivers, significantly influences the child’s experience and cooperation during surgery.

Another area that requires further exploration is the long-term impact of speech therapy intervention in pediatric ABS. Given the neuroplasticity of the pediatric brain, early and targeted intervention can lead to better recovery and adaptation compared to adults.

This study addresses the research gap concerning the role of speech therapy in pediatric ABS. By examining both the Moroccan context and global practices, the main objective is to explore and define the recommended SPL approach for pediatric ABS. This includes assessing current practices, identifying challenges, and evaluating outcomes to improve care protocols in Morocco and ensure that pediatric patients undergoing ABS receive tailored and effective interventions.

The central research question guiding this study is as follows: What is the recommended SLP approach for pediatric ABS to optimize preoperative preparation, intraoperative assessment, and postoperative recovery?

## Materials and methods

​​​​This exploratory study aims to explore and describe speech therapy interventions during pediatric ABS in Morocco and internationally. Focused on University Hospitals with active ABS practices, the study seeks to understand current approaches, identify challenges, and assess outcomes to improve care and protocols. Combining research and expert perspectives highlights the unique aspects of pediatric ABS and the tools used to evaluate language and cognitive functions.

Ethical approval

The Ethics Committee for Biomedical Research (N°C64/20) approved the study and adhered to the Declaration of Helsinki principles.

Study population

The study targeted healthcare professionals from 11 teams involved in pediatric ABS: Morocco, Neurosurgery department at Hôpital des Spécialités de Rabat, CHU ibn sina; France, CHU de la Timone (Marseille), Hôpital Sainte-Anne (Paris), Hôpital de Kremlin-Bicêtre, CHU d'Angers, and CHU de Lyon; Algeria, CHU Mustapha-Bacha; Canada, Montreal Hospital; Switzerland, a private clinic; Colombia, Clinica Foscal; Greece, a leading pediatric neurosurgery center.

Sampling strategy

The study employed a purposive sampling method to select 11 international teams with diverse geographical and professional expertise in pediatric ABS. This approach was chosen to capture a broad spectrum of practices and perspectives, enhancing the study’s external validity. The selected teams included healthcare professionals such as neurosurgeons, speech therapists, neuropsychologists, nurses, physiotherapists, anesthetists, and radiologists, all of whom voluntarily agreed to participate.

Inclusion Criteria

Professionals directly involved in pediatric ABS, including SLTs, neurosurgeons, and other relevant professionals.

Data collection

Data collection took place from November 2021 to June 2022.

Survey

The study utilized a web-based survey to capture the clinical practices of 11 pediatric awake surgery teams. The user-friendly tools were designed to be accessible from both computers and smartphones, ensuring ease of use. Three questionnaires tailored for neurosurgeons, speech therapists, and other professionals (e.g., anesthetists and neuropsychologists) were developed and translated into French, English, and Spanish to address the diverse specialties involved in awake surgery and the international reach of the survey.

The survey was conducted over four months, from March to June 2022, as part of a study exploring and describing speech therapy interventions during pediatric ABS. Participants were strategically recruited through professional speech-language therapy (SLT) and neurosurgery networks, focusing on those involved in ABS, and through targeted outreach on professional social media platforms (LinkedIn, Instagram, Facebook, and WhatsApp). These platforms were selected for their professional reach and ability to connect with relevant SLT and neurosurgery specialists. The survey ensured anonymity and confidentiality to encourage candid responses. By completing and submitting the survey, participants provided implied consent for their responses to be stored and used for research purposes. No financial or other incentives were provided.

Survey design: The survey instrument used in this study was developed to capture detailed insights into the practices of speech therapists and other professionals involved in pediatric ABS. The design process followed established guidelines by Eysenbach (2004), incorporating demographic information, clinical practices, challenges, and outcomes. It involved a thorough literature review and consultations with subject matter experts in neurology, neurosurgery, speech therapy, and pediatric care.

The survey underwent a pilot phase with a small group of experts to ensure clarity, validity, and comprehensiveness. Based on their feedback, revisions were made to enhance its usability and relevance. The final survey included both closed and open-ended questions, enabling participants to provide comprehensive feedback.

Case Study

The study population includes three children who underwent ABS in Morocco between 2017 and 2022. Data for this study were meticulously collected by systematically reviewing the medical records of these pediatric patients at the Neurosurgery Department of the Hôpital des Spécialités de Rabat. With patient consent, specific medical records sections, including preoperative, perioperative, and postoperative evaluations, were carefully examined, focusing on the SPL interventions and outcomes.

The study included three pediatric cases from Morocco because this represents the pediatric ABS procedures performed at the selected center within the specified timeframe. While this is a small sample size, it reflects the limited adoption of ABS in pediatric populations in Morocco due to its highly specialized nature and the challenges involved in performing awake surgeries in children.

Key data points such as patient demographics, clinical presentation, surgical details, and outcomes were extracted, along with the analysis of relevant imaging results and medical conclusions; the extracted data were verified for accuracy, ensuring completeness and consistency while maintaining patient confidentiality and adhering to ethical guidelines. This study aims to synthesize critical information from these cases to enhance our understanding of pediatric ABS, particularly in comparison to adult procedures, and to identify variations in speech therapy approaches.

Case selection criteria: The sample size was naturally constrained by the specialized nature of ABS and its relatively recent implementation in Morocco. Although the small sample size limits the generalizability of the findings, it offers valuable insights into a unique and underexplored area.

Inclusion criteria: Pediatric patients (aged ≤18 years) who underwent ABS between 2017 and 2022 at the Neurosurgery Department of Hôpital des Spécialités in Rabat, with complete medical records documenting preoperative, intraoperative, and postoperative evaluations, including SLP interventions. Parental or legal guardian consent to review medical records and include anonymized data in the study.

Exclusion criteria: Incomplete medical records or absence of documentation on speech therapy interventions; cases where the surgery was aborted or transitioned to conventional brain surgery due to unforeseen circumstances; and patients with significant comorbidities unrelated to the neurological condition that could independently affect language or cognitive outcomes.

Case study assessment tools

The MT86 (Protocole Montréal-Toulouse 86) and DO80 (Dénomination d’Objets en 80 Images) were specifically adapted for evaluating speech and cognitive functions in pediatric patients undergoing ABS at Hôpital des Spécialités in Rabat. These tools, widely used in French-speaking clinical settings, were selected for their proven effectiveness in assessing language functions and their suitability for the Moroccan context.

MT86 (Nespoulous et al., 1986) assesses language comprehension, naming, repetition, and fluency. In 2003, the neuropsychology team at Hôpital des Spécialités translated the MT86 into Arabic, adapting it to the linguistic and cultural characteristics of Morocco to improve its relevance for pediatric evaluations.

DO80 (Deloche & Hannequin, 1997) evaluates naming skills using image identification. Its pictorial format required no linguistic or cultural modification, making it straightforward for use in the Moroccan context.

Phases of Administration

Preoperative: Establish baseline cognitive and language abilities to guide surgical planning.

Intraoperative: Monitor language functions in real-time to preserve critical areas during surgery.

Postoperative: Reassess cognitive and language abilities to support rehabilitation planning.

The integration of these tools, tailored to Morocco's cultural and clinical setting, highlights their critical role in ensuring the safety, precision, and success of pediatric ABS procedures.

Data analysis

Quantitative Analysis

Data were analyzed using IBM SPSS Statistics version 25 (IBM Corp., Armonk, NY). Descriptive and inferential statistical techniques were applied as follows:

Descriptive statistics: Frequencies, percentages, and means were used to summarize demographic information and key variables related to pediatric ABS practices. Charts and graphs were generated to facilitate visual interpretation.

Inferential statistics: These were conducted to explore key relationships within the data. Tests were selected based on the study's objectives and data characteristics. For example, Chi-square tests were applied to examine relationships between categorical variables, such as the use of specific SLT tools across different countries.

Qualitative Analysis

Content analysis was performed on survey responses and patient records using a coding framework informed by existing literature and the study's objectives. The analysis focused on the following themes: the role of SLPs in ABS, including training and preparation for intraoperative care; managing risks associated with SLP during pediatric ABS; assessing patient outcomes related to language and cognitive mapping; and fostering interdisciplinary collaboration during ABS.

Two independent researchers coded the data using a pre-determined schema. Discrepancies were resolved through discussion, achieving an inter-rater reliability of 90%. This triangulated approach ensured robustness and consistency in data interpretation.

Literature review

Comprehensive bibliographic searches were conducted using NCBI, ScienceDirect, PubMed, and Google Scholar. This ensured a thorough exploration of available literature across digital and print platforms to contextualize findings and identify best practices in pediatric ABS.

Ethical considerations

Stringent measures were implemented to safeguard the rights and freedoms of the professionals and patients involved, including free and informed consent, anonymity and confidentiality, transparency in data analysis, and fairness in participant treatment and result interpretation.

Study design note

This study does not include a randomized controlled trial (RCT). Therefore, the CONSORT checklist and flow diagram are not applicable.

## Results

Our investigation into speech therapy interventions during awake pediatric brain surgery incorporated data from a range of international institutions, including France, Canada, Algeria, Switzerland, Colombia, Greece, and Morocco. Additionally, we reviewed medical records for three pediatric patients who underwent ABS at the Hopital des Spécialités in Rabat, Morocco. 

Respondent identification

Table 1 presents the distribution of respondents by years of practice and specialty, revealing that a significant portion of the surveyed professionals have over five years of experience, indicating a sample of seasoned practitioners. The high number of SLPs and neurosurgeons reflects stability in these specialties, despite the noted shortage of pediatric neurosurgeons. 

**Table 1:** Distribution of respondents by years of practice and specialty

The respondent's specialties	Years of practice			
	<5 years	=5 years	>5 years	Total (N=61)
Neurosurgeon	0	1	14	15
Pediatric neurosurgeon	2	0	0	2
Speech therapist	5	0	23	28
Anesthetist	1	2	7	10
Neuropsychologist	1	2	3	6
Total (N=61)	9	5	47	61

Table 2 illustrates that there is a strong preference for working in the public sector among respondents. This preference may be influenced by job opportunities, personal preferences, or specific labor market conditions within these specialties. 

**Table 2:** Specialties of respondents and the sectors in which they are employed

The respondent's specialties	The sector of work		Total (N=61)
	Public	Private	
Neurosurgeon	13	2	15
Pediatric neurosurgeon	0	2	2
Speech therapist	20	8	28
Anesthetist	8	2	10
Neuropsychologist	5	1	6
Total (N=61)	46	15	61

Training in awake brain surgery

Table 3 details the duration of training in ABS and the countries where this training took place, indicating that most training occurs in France and typically lasts less than three months. This highlights the need to raise awareness about the importance of awake surgery and brain mapping training. 

**Table 3:** Duration of training in ABS and the countries where the training took place ABS: awake brain surgery

Countries of ABS training	The duration of training in ABS					Total (N=61)
	1 year	6 months	3 months	>1 year	<3 month	
France	6	5	6	5	24	46
USA	0	0	0	1	0	1
Morocco	0	0	0	2	1	3
Canada	0	0	0	1	0	1
Switzerland	1	1	0	1	0	3
Algeria	0	1	0	1	1	3
Greece	0	0	3	0	1	4
Total (N=61)	7	7	9	11	27	61

According to Table 4, all respondents regard awake surgery training as fundamental; however, there is a gap between this recognition and the actual receipt of such training. Specifically, 34 respondents (55.7%) have received training in awake surgery, while 27 respondents (44.3%) have not. 

**Table 4:** Perceived importance of training in ABS among the respondents ABS: awake brain surgery

Training in awake surgery	Yes	No	Total (N=61)	Percentage (%)
Fundamental training	34	27	61	100%

The awake brain surgery

Table 5 provides an overview of pre-surgical warning symptoms and possible after-effects of surgery. It reveals that 18% of the warning symptoms involved headaches, 44% involved convulsions or seizures, and 21% involved slurred speech. Post-surgery, 34% of cases experienced tumor recurrence, 23% had convulsions or epileptic seizures, 26% had neurolinguistic disorders, and 16% had motor disorders. These findings underscore the critical need for thorough pre-surgical assessments and tailored interventions to manage potential complications and enhance patient recovery. 

**Table 5:** Preliminary symptoms of a brain tumor and the potential complications after ABS ABS: awake brain surgery

Warning symptoms	Possible after-effects of surgery				Total (N=61 respondents)	Percentage (%)
	Tumor recurrence	Convulsions or epileptic seizures	Neurolinguistic disorders	Motor disorders		
Headaches	5	0	5	1	11	18.03%
Convulsions or seizures	15	4	0	8	27	44.26%
Slurred speech	1	0	11	1	13	21.31%
Intracranial hypertension	0	10	0	0	10	16.39%
Total (N=61)	21	14	16	10	61	100%
Percentage (%)	34.43%	22.95%	26.23%	16.39%	100%	

Table 6 highlights that most patients undergoing awake surgery are over 10 years old (62.3% of cases), likely due to better cooperation and understanding in older children. This emphasizes the importance of training neurosurgeons in pediatric brain surgery for younger children and adapting brain mapping techniques to effectively assess cognitive skills across different age groups.

**Table 6:** Age distribution of patients undergoing awake surgery

The average age	N (Respondents)	Percentage	Valid percentage	Cumulative percentage
3-5 years	6	6.0%	9.8%	9.8%
7-9 years old	13	13.0%	21.3%	31.1%
More than 10 years	38	38.0%	62.3%	93.4%
No strict age criterion	4	4.0%	6.6%	100.0%
Total (N=61)	61	61.0%	100.0%	

Speech therapy role and assessment

Table 7 highlights the distribution of psychological support provided preoperatively and postoperatively. The majority of respondents view the role of the speech therapist as primordial (primordial, 59 out of 61) for assessing and rehabilitating patients' linguistic and cognitive functions during ABS. This emphasizes the need to bolster the presence and training of SLPs, and there is a greater need for psychological support preoperatively than postoperatively, to maximize recovery chances and maintain a good quality of life, as children may struggle more with understanding and managing the implications of awake surgery.

**Table 7:** Distribution of psychological support provided preoperatively and postoperatively, and the perceived importance of the speech therapist's role

The importance of the speech therapist's role	The psychological support		Total (N=61)	Percentage (%)
	preoperative	postoperative		
Primordial	44	15	59	95.77%
Optional	2	0	2	3.28%
Total (N=61)	46	15	61	100%

Table 8 summarizes the reasons for failing to assess written language components. Factors such as patient position, time constraints, and lack of suitable tools were identified, and each factor received a mean score of 1.0000, indicating unanimous agreement among the respondents. The standard deviation of 0.00000 across all factors suggests no variability in responses, reinforcing the consistency of these reasons as barriers to assessing written language components. Developing specialized tools to facilitate comprehensive and accurate evaluations is crucial to ensuring essential language functions are assessed and protected.

**Table 8:** Assessment of written language, including mean values for unassessed components Mean score (0-1): The values represent binary scores (0 or 1), where 1 indicates a failure to assess the corresponding component. Std. deviation: The standard deviation for each score is 0.00000, indicating that all respondents gave the same score (1). Range (0-1): The possible range of scores, from 0 (not assessed) to 1 (failure to assess).

Failure to assess written language	Mean score (0-1)	N (respondents)	Std. deviation	Range (0-1)
Patient position	1.0000	10	0.00000	0-1
Time	1.0000	10	0.00000	0-1
No suitable tools	1.0000	8	0.00000	0-1
Total (N=28)	1.0000	28	0.00000	0-1

Table 9 highlights the tools used by speech therapists for brain mapping and the functions assessed. The tools include "DO80" (Dénomination Orale d'images) "BDAE", "VOL du PC" (Évaluation fonctionnelle de la lecture. Chez les sujets de 11 à 18 ans) and "L2MA",(Batterie Langage oral, Langage écrit, Mémoire, and Attention) with a clear preference for tools that assess comprehension and memory. Additionally, SLPs play a crucial role in evaluating various brain functions throughout all stages of the procedure, including functions such as speech, comprehension, memory, praxis, and executive functions.

**Table 9:** Tools used by speech therapists for brain mapping and the functions to be assessed D80: Dénomination Orale d'images 80; BDAE: Boston Diagnostic Aphasia Examination; L2MA: Batterie Language Oral, Language Écrit, Mémoire, and Attention

Functions to be assessed in brain mapping	The tools used by the speech therapist for brain mapping				Total (N=28)	Percentage (%)
	D80	BDAE	VOL du PC	L2MA		
Speech	0	4	0	3	7	25%
Comprehension	7	0	0	0	7	25%
Memory	2	5	0	0	7	25%
Praxis	1	0	4	0	5	17.86%
Executive functions	0	0	2	0	2	7.14%
Total (N=28)	10	9	6	3	28	100%
Percentage (%)	35.71%	32.14%	21.43%	10.71%	100%	

According to Table 10, assessments are predominantly conducted in the patient's mother tongue and often through digital formats, which is crucial for accurate evaluations. 

**Table 10:** Languages and format used for SLPs assessment during brain mapping SLPs: speech-language pathologist

Test presentation	Test language			Total (N=28)	Percentage (%)
	Mother tongue	Acquired language	Both languages		
In Paper	0	0	2	2	7.14%
Digital	17	3	6	26	92.86%
Total (N=28)	17	3	8	28	100%
Percentage (%)	60.71%	10.71%	28.57%	100%	

The duration of speech assessments varies, with most taking around two hours as mentioned in Table 11. This data emphasizes the necessity of comprehensive testing across all cognitive-linguistic functions, to address individual patient needs and ensure thorough evaluations. Adopting a holistic approach is vital for optimizing post-surgical outcomes and enhancing the quality of life for young patients.

**Table 11:** Maximum time allocated for brain mapping

Timing of speech assessment	Maximum time for brain mapping				Total (N=28)
	less than 1 hour	2 hours	more than 2 hours	varies from case to case	
During the 3 Stages	7	14	2	5	28
Percentage (%)	25%	50%	7.14%	17.86%	100%

Table 12 demonstrates the Pearson correlation between possible after-effects of surgery and speech therapy intervention and the follow-up. There is a significant positive correlation between these variables. Approximately 80% of cases benefit from systematic speech therapy, indicating healthcare professionals' strong belief in its crucial role in recovery and well-being after surgery. The potential complications after surgery include tumor recurrence (35%), neurolinguistic disorders (30%), seizures (20%), and motor disorders (15%). These statistics highlight the need for comprehensive medical follow-up and various rehabilitative therapies to optimize recovery and improve the quality of life for patients.

**Table 12:** Pearson correlation between possible after-effects of surgery and speech therapy intervention and the follow-up ^**^Correlation is significant at the 0.01 level (2-tailed).

		Possible after-effects of surgery	Speech therapy intervention and the follow-up after surgery
Possible after-effects of surgery	Pearson correlation	1	0.340^**^
	Sig. (2-tailed)		0.007
	N	61	61
Speech therapy intervention and the follow-up after surgery	Pearson correlation	0.340^**^	1
	Sig. (2-tailed)	0.007	
	N	61	61

Presentation of three pediatric patients undergoing awake brain surgery in Morocco

This section details three pediatric cases of ABS in Morocco, focusing on the SLP interventions and outcomes. We extracted data from medical records in the Neurosurgery Department at the Hôpital des Spécialités de Rabat for cases that occurred during the period between 2017 and 2022. Table 13 provides a detailed overview of these cases, including demographic information, clinical presentation, and surgical experiences. It covers aspects such as preoperative psychological preparation, intraoperative assessments, and postoperative results, highlighting the role of SLPs and any challenges encountered during the procedure.

**Table 13:** Detailed case data on patients who underwent ABS, including their surgical experience and outcomes ABS: awake brain surgery; MT86: Montréal-Toulouse 86; DO80: Dénomination Orale d'images 80

	Patient 1	Patient 2	Patient 3
Year of ABS	2017	2017	2017
Age	8 years	11 years	13 years
Gender	Female	Female	Female
Educational level	CE4 (fourth-grade elementary education in Morocco)	CE6 (sixth-grade elementary education in Morocco)	First-year middle school
Handedness	Right-handed	Right-handed	Right-handed
Initial symptoms	Convulsive seizure	Convulsive seizure	Convulsive seizure
Type of lesion/location	Low-grade glioma	Low-grade glioma	Low-grade glioma
Duration of psychological preparation	3 preoperative sessions	3 preoperative sessions	3 preoperative sessions
Psychological preparation interveners	Speech therapist	Speech therapist	Speech therapist
Explanation tools	Videos and explanations	Videos and explanations	Videos and explanations
Preoperative assessment by speech-language therapist	_	_	_
Assessment tools	MT86, DO80	MT86, DO80	MT86, DO80
Preoperative deficits	None	None	None
Patient experience preoperatively	_	_	_
Intraoperative assessment by speech language therapist	Spontaneous language, automatic language	Spontaneous language, automatic language	Spontaneous language, automatic language
Assessment tools	DO80	DO80	DO80
Intraoperative deficits	None	None	None
Duration of operation	4 to 6 hours	4 to 6 hours	4 to 6 hours
Challenges encountered	Panic, fatigue	Panic, fatigue	Panic, fatigue
Experience during operation	Normal, except for fatigue	Normal, except for fatigue	Normal, except for fatigue
Postoperative assessment by speech language therapist	_	_	_
Assessment tools	MT86	MT86	MT86
Postoperative deficits	Asymptomatic	Asymptomatic	Asymptomatic
Experience postoperation (difficulties-impact of ABS)	None	None	None
Post-surgical sequelae	None	None	None
Surgical reintervention	Never, benign tumor case	Never, benign tumor case	Never, benign tumor case
Postoperative speech language therapist rehabilitation	Never	Never	Never
Complementary treatment	Antiepileptic	Antiepileptic	Antiepileptic
Family support	_	_	_
Observations	_	_	_

Patient Overview

The study presents three pediatric patients, all female and right-handed, who underwent ABS in 2017 for low-grade gliomas. Their ages ranged from 8 to 13 years, and they were at different educational levels: CE4, fourth grade elementary (Patient 1), CE6 (Patient 2), and first year of middle school (Patient 3). The initial symptoms for all three patients were convulsive seizures, which led to the discovery of the tumors. This mirrors findings from Herta J et al. study, where seizures were the main presenting symptom for all patients and the most common pathology observed was ganglioglioma, found in 27% of patients, most lesions were in language-related areas [20].

Psychological Preparation

All three patients received psychological preparation involving three preoperative sessions conducted by a speech therapist. This consistent approach highlights the importance of psychological readiness for young patients undergoing ABS. The use of videos and explanations as preparatory tools was key in helping the patients understand the procedure, likely reducing anxiety and improving cooperation during surgery [21].

Preoperative and Intraoperative Speech Therapy Assessment

The preoperative assessment included tools such as the MT86 and DO80, with no identified deficits in any of the patients. During surgery, the speech therapist assessed spontaneous and automatic language using the DO80 tool. None of the patients exhibited intraoperative deficits, which might suggest that the awake surgery was successful in preserving their language functions. However, it is notable that the patients experienced challenges such as panic and fatigue during the operation, which lasted between four to six hours, indicating that even with thorough preparation, the reality of the surgery can be overwhelming.

Postoperative Outcomes

Postoperatively, the patients remained asymptomatic, with no surgical reintervention required, and no postoperative speech therapy rehabilitation was necessary. This outcome is encouraging, as it suggests that the surgery did not result in any immediate or observable long-term cognitive or speech deficits. Regular assessments might be essential to detect any subtle deficits that could emerge over time.

Family Support and Observations

Family support is not detailed in the provided data, but it plays a crucial role in the recovery and overall well-being of pediatric patients. Including more information on family involvement and the support systems in place would provide a more comprehensive view of the patient's recovery journey. Additionally, observations regarding the patients' adaptation to post-surgery life, their psychological state, and academic performance would contribute valuable insights [21].

## Discussion

This section delves into the findings from our comprehensive review of speech therapy interventions during pediatric ABS. The study integrates data from 11 international institutions, focusing on practices, training, and tools, alongside a case study from the Hopital des Spécialités in Rabat, Morocco. This review aims to offer insights into current methodologies and identify gaps in practice.

The data reveals that most respondents have over five years of experience (Table 1), indicating a sample composed mainly of seasoned professionals. This experience distribution suggests a level of expertise within the respondent pool that lends credibility to the findings, reflecting the perspectives of those well-versed in their respective fields. Moreover, the range of experience across specialties highlights the importance of collaborative practice, where different experts come together to offer comprehensive care, especially in the context of the complex procedures associated with ABS.

The dominance of SLPs (28 respondents) and neurosurgeons (15 respondents) highlights the stability and significance of these specialties in the context of ABS. However, the scarcity of pediatric neurosurgeons (only two respondents) points to a concerning shortage in a critical area of specialty, suggesting an area where further training and recruitment may be necessary.

Regarding the work sector, there is a notable preference for the public sector across all specialties, particularly among neurosurgeons and speech therapists. This trend might be driven by the availability of opportunities, job security, or specific labor market demands in these fields, as illustrated in Table 2. This preference for the public sector may also reflect a more structured system for surgical training and patient management, which is vital for the delicate nature of ABS.

Training in ABS and brain mapping is predominantly conducted in France, with most training durations being under three months. Table 3 highlights this training concentration in France and the relatively short duration, which may reflect the highly structured and well-regarded programs available there. The early adoption of awake oncology surgery in France, pioneered by Peggy Gatignol, has contributed to the prevalence of training in this region.

While French training programs are prominent, the absence of similarly structured programs in Morocco and other regions may explain the existing gaps in ABS training; this is reflected in the 44.3% of respondents who reported lacking formal ABS training. Despite unanimous agreement among respondents on the fundamental importance of ABS training, this gap underscores a significant disparity between recognizing its value and its actual availability. It also emphasizes the urgent need to expand access to these programs, particularly in regions where such skills are less commonly taught.

The critical need for specialized training for SLPs involved in awake craniotomy is further highlighted by a UK survey, where 96% of SLPs advocated for structured training and the value of peer support networks; this finding aligns with our study's emphasis on the role of collaborative learning and support in enhancing SLP practice. The gap in access to specialized training programs, especially outside of France, requires attention, underscoring the need for international collaborations and investments to expand the availability of ABS training in regions with less established infrastructure.

Seizures, headaches, slurred speech, and intracranial hypertension are critical warning symptoms that help the surgical team anticipate potential complications. These symptoms, along with observed post-surgical effects such as tumor recurrence (34%), neurolinguistic disorders (26%), and motor disorders (16%), emphasize the importance of comprehensive pre-surgical assessments and tailored interventions (Table 5).

Integrating detailed preoperative evaluations with vigilant postoperative care can help mitigate risks and improve patient outcomes. In particular, the role of SLPs in preoperative cognitive and communication assessments is crucial for identifying potential risks and informing surgical strategies. A UK survey further supports the importance of preoperative evaluations, which found that nearly all SLPs (96%) consider preoperative evaluation critical and actively assess service users before awake craniotomy [15]. However, while most SLPs emphasize the importance of these evaluations, the practice’s thoroughness varies across regions. Almost all practitioners in the UK conduct these assessments routinely, but access to comprehensive preoperative evaluations may be limited in lower-resourced areas.

The data also indicates that most awake surgeries are performed on children over 10 years old (62.3%), likely due to older children's greater cooperation and understanding. Plus, contraindications, such as age-related factors, tumor type, and brain maturity, were highlighted by different teams. Table 6 highlights the age distribution of patients undergoing awake surgery and underscores the necessity of developing specialized training and techniques to effectively manage younger patients, particularly those aged three to nine years, who may require different approaches due to their developmental stages. Lohkamp L-N reported similar findings, noting that ABS is as feasible, safe, and effective in children as it is in adults [7]. However, certain age-specific factors and adaptations are crucial. Extensive preoperative evaluations, including psychological and neuropsychological assessments, are essential for the procedure's success.

The role of speech therapists in ABS is overwhelmingly seen as crucial, with 96.7% of respondents considering it primordial preoperatively and postoperatively (Table 7). The majority of speech therapists involved in these interventions are women (85.7%), a trend consistent with the broader gender distribution in SLP, where women typically make up the majority of the workforce. Various sociocultural factors, including gender roles and career preferences, contribute to this distribution. Additionally, 70% of these professionals work in university hospitals (UH) such as Lyon, Kremlin-Bicêtre, Angers, Mustapha Bacha, Lausanne, Athens, and the University Hospital of Rabat. They operate across both the public (75%) and private sectors (25%), underscoring their vital role in the success of ABSs. The greater emphasis on preoperative psychological support reflects the challenges children may face in coping with the implications of awake surgery. This highlights the need for a strong, multidisciplinary approach to pediatric brain surgery that includes robust support systems for patients and their families. A retrospective review and prospective outcome analysis of pediatric patients with CNS lesions undergoing ABS from 2005 to 2018 was conducted at the University of Lyon, France [7]. Their findings emphasized that psychological support and meticulous preoperative preparation were crucial, especially for younger patients. Despite the inherent challenges of ABS in children under 10 years old, the procedure was generally well-tolerated, with no significant psychological or neurological deterioration reported. Postoperative rehabilitation, especially involving speech therapy, plays a vital role in ensuring the continued success of the procedure, with many patients requiring ongoing therapy to maintain language and cognitive functions.

Further insights into assessment methodologies reveal a nuanced approach tailored to individual patient needs. Table 9 provides an overview of the assessment tools used, which cover a comprehensive range of cognitive functions, including attention, executive function, memory, language components (such as naming, comprehension, and repetition), and behavioral assessments. These evaluations varied across teams but consistently emphasized holistic patient assessments during the preoperative, intraoperative, and postoperative phases.

Assessment tools and batteries were chosen based on age-appropriate criteria and developmental milestones. This approach aligns with the study by O'Neill et al. [15], where commonly used assessments included the Boston Naming Test (63%) and the Comprehensive Aphasia Test (50%). Informal methods were also employed, such as observing communication and conducting oral-motor examinations.

The child's age significantly influences the assessment process, as younger patients may require additional adaptations to standard assessments to ensure their comfort and cooperation during the procedure. However, the Moroccan cases revealed a more standardized approach to speech therapy interventions than international practices. The Moroccan team employed uniform assessment procedures without tailoring them to the individual patient's age, linguistic abilities, or educational level. They reported using standardized assessments but noted that cultural and linguistic adaptations were often overlooked. This highlights the critical need for incorporating culturally sensitive tools to ensure more accurate evaluations in diverse populations.

The presented cases highlight the integral role of SLP in pediatric ABS, where precise language mapping and preservation are paramount. Psychological preparation and thorough preoperative assessments facilitated effective intraoperative monitoring, leading to positive postoperative language outcomes. The minimal need for postoperative speech therapy underscores the effectiveness of proactive SLPs' involvement in optimizing language function. Interdisciplinary collaboration remains essential in optimizing patient care and rehabilitation strategies across diverse clinical settings. This aligns with the UK practice where postoperative review is standard but varies in thoroughness and highlights the importance of continuity of care, as noted by 58% of SLPs experiencing inconsistent speech therapy involvement across different phases [15].

Furthermore, the case study (Table 13) demonstrates a successful outcome in preserving language function and avoiding postoperative complications. However, the study could be enriched by more detailed accounts of patient experiences, family support, and long-term follow-up. While teams uniformly stressed the importance of postoperative rehabilitation and therapeutic education for patients and families, particularly in pediatric cases, parental involvement and psychological support were crucial throughout the surgical journey. Due to the anticipated increased psychological sensitivity and the complex age-related factors in children, there is a considerable gap in applying this technique to pediatric cases. The primary obstacle is the need for thorough psychological evaluation and individualized patient preparation, which could delay urgent lesion removal, especially in cases with aggressive malignancies. Only a handful of studies have specifically examined the use of ABS in children [7].

Most respondents agreed on the importance of evaluating written language components, particularly in pediatric brain mapping during awake surgery. This consensus highlights the necessity of preserving academic skills, such as reading, during and after surgical procedures. However, the assessment of written language components in ABS is hindered by several consistent barriers: patient positioning, time constraints, and the lack of suitable tools, as mentioned in Table 11. The unanimous agreement among respondents on these factors underscores the need to develop specialized tools and methodologies to overcome these challenges and ensure that essential language functions are adequately assessed.

In brain mapping, there is a clear preference for digital tools and colloquial language to ensure accurate results (Table 13). This preference points to the need for further development and optimization of digital tools that are tailored to different languages and can accommodate the specific needs of each case. Moreover, the involvement of SLPs in assessing various brain functions throughout the procedure is critical, especially in ensuring a comprehensive evaluation that includes less frequently assessed functions like praxis and executive functions.

The significant positive correlation between the potential after-effects of surgery and the need for speech therapy intervention and follow-up, as shown in Table 7, further underscores the essential role of speech therapy in the recovery process. This finding emphasizes the critical role of SLPs within the multidisciplinary care team, not only in addressing immediate language issues but also in ensuring long-term cognitive and emotional recovery for pediatric patients. The high percentage of cases requiring speech therapy (80%) following surgery highlights the strong consensus among healthcare professionals regarding the importance of this intervention in optimizing patient outcomes and quality of life. This correlation suggests that as the incidence of complications increases, so does the reliance on speech therapy, stressing the need for comprehensive post-surgical rehabilitation plans that include speech therapy as a core component.

However, this study has several limitations to consider when interpreting the findings. Cultural differences and variability in healthcare systems across the respondents' locations may have influenced their practices and perspectives, potentially affecting the comparability of results. For example, countries with established training programs may demonstrate different outcomes compared to regions where such training is less accessible. Additionally, the overrepresentation of countries with advanced ABS programs and the underrepresentation of lower-resource regions may have introduced bias into the results. It is crucial to interpret these findings with an understanding of the varying access to specialized training and resources across regions, which could impact the generalizability of the data. This underscores the need for further research that includes a more diverse sample of areas, particularly lower-resource settings, to understand these findings' global applicability better.

Despite these limitations, the study offers valuable insights into current practices, challenges, and opportunities for speech therapy in pediatric ABS, laying a foundation for future research and clinical advancements.

Despite these limitations, the study offers valuable insights into current practices, challenges, and opportunities for speech therapy in pediatric ABS, laying the groundwork for further research and clinical advancements. As the first study of its kind in Morocco, it explores the critical role of speech therapy in pediatric ABS, addressing a gap that has previously been overlooked in the literature. Furthermore, the study provides a unique opportunity to compare international practices, revealing commonalities and regional variations in ABS procedures. It aims to influence the future of SLP practice and research, especially in regions where ABS is emerging, but SLP training for such interventions remains limited. By examining current practices, challenges, and outcomes, the study seeks to contribute to developing tailored interventions, enhanced training programs, and improved patient care strategies.

In summary of the discussion, this study underscores the significance of team composition, training background, and procedural methodologies in awake pediatric brain surgery. It highlights the essential role of specialized training, thorough pre-, intra-, and post-surgical assessments, and the integral involvement of speech therapy in improving patient outcomes. Addressing the gaps identified in training, resource availability, and multidisciplinary support is crucial for advancing care in this specialized field. Moving forward, research should evaluate the feasibility of implementing structured ABS training programs, particularly in regions like Morocco, to bridge existing gaps and standardize practices in pediatric brain surgery.

## Conclusions

Our research provides preliminary insights into the challenges and nuances of pediatric awake surgery, particularly within speech therapy interventions, and highlights the need for further research to refine tailored approaches that address the diverse needs of pediatric patients undergoing awake neurosurgical procedures. This is especially crucial in resource-limited settings like Morocco. The findings underscore the evolving nature of pediatric neurosurgical practices and emphasize the importance of enhancing interdisciplinary collaboration and patient-centered care. As the first study in Morocco to explore the role of speech therapy in this domain, it fills a critical gap in the literature. It engages a broad audience, including neurosurgeons, speech therapists, and other stakeholders involved in awake surgery. The survey data from our study highlight the perceived importance of speech therapy (SLP) in these cases, reinforcing the need for further integration into neurosurgical practices. However, while offering insights into short-term patient outcomes, the case study data are based on a small sample size and should be considered as preliminary evidence. These outcomes require validation through larger cohorts to draw more definitive conclusions.

We also encountered several challenges, such as the limited literature on pediatric awake surgery and the scarcity of surgical teams practicing this technique in Morocco. These constraints limited our access to patient records and impacted the depth of our case study evaluation. As a result, while the findings suggest potential benefits from speech therapy interventions, the small sample size and limited geographic scope restrict the generalizability of the conclusions. Long-term follow-up data is needed to substantiate claims regarding the sustained benefits of SLP interventions in this context. The observed variability in outcomes and practices can likely be attributed to factors such as the lack of pediatric-specific tools and limited training among surgical teams. These challenges highlight significant gaps in the current approach to pediatric ABS and neurolinguistic assessment. Given these findings, we recommend developing pediatric-specific tools and training programs for speech therapy in neurosurgery settings. This will require ongoing research and collaboration among stakeholders, including neurosurgeons, speech therapists, and policymakers. This study serves as a starting point for further research, and we emphasize the need for future studies to expand the sample size and incorporate long-term data collection. By fostering a systematic approach to tool development and training, we can enhance the quality of care for pediatric patients undergoing awake neurosurgical procedures and optimize their outcomes. Regular, systematic research studies are essential for monitoring, evaluating, and advancing practices in pediatric ABS and brain mapping, ensuring continuous improvement in this critical area of healthcare.

## Appendices

Survey for neurosurgeons

We are conducting research on " Speech-Language Pathology Intervention in Pediatric Awake Brain Surgery: A Global Perspective."

Due to the limited number of professionals involved in pediatric awake brain surgery in Morocco, our survey also reaches an international scale. The objective is to better understand the practices of SLPs and other professionals working in this specific field.

For this purpose, we invite you to answer this questionnaire designed for professionals (speech-language pathologists, neurosurgeons, neuropsychologists, anesthesiologists, physical therapists, psychomotor therapists, and psychologists) involved in pediatric awake brain surgery. Your responses will be invaluable to enhance our research. We assure you that your answers will be treated with confidentiality and anonymity. We appreciate the time and attention you dedicate to this survey.

I. Demographic and Professional Data:

Are you:

General Neurosurgeon

Pediatric Neurosurgeon

In which country do you practice?

Morocco

France

Spain

United States of America

Lebanon

Tunisia

Algeria

Canada

Belgium

Italy

United Arab Emirates

Other (please specify):

How long have you been practicing?

Less than 5 years

5 years

More than 5 years

What is your work sector?

Public

Private

Where was your initial training?

Public

Private

In Morocco

Abroad

In which institution do you practice?

II. Speech Therapy Intervention in Pediatric Awake Brain Surgery:

Have you received training in awake surgery and/or brain mapping?

Yes

No

→ If yes:

In what setting?

Initial training

Continuing education

Personal initiative

Where did you receive this training?

France

Spain

USA

Italy

Mexico

Morocco

Lebanon

Algeria

Tunisia

Other (please specify):

Who provided your training, if possible?

Duration of the training?

1 month

3 months

6 months

1 year

2 years

Other (please specify):

→ If no, are you willing to receive training on this?

Yes

No

Do you think this training is:

Essential

Useful but optional

Should be mandatory

How many years have you practiced awake brain surgery?

Less than 5 years

5 years

More than 5 years

More than 10 years

In your experience, which professionals should ideally be involved in awake brain surgery?

Neurosurgeon

Anesthesiologist

Speech-Language Pathologist

Psychomotor Therapist

Physical Therapist

Psychologist

Neuropsychologist

Other (please specify):

Do you practice pediatric awake brain surgery?

Yes

No

→ If no, why not?

Lack of training

Not specialized in pediatric neurosurgery

Complexity of the pediatric brain

High risk of complications

Other (please specify):

→ Are there neurosurgeons specialized in pediatric brain surgery?

Yes

No

→ If yes:

In which institution?

How many children have you operated on so far?

When did you start performing awake surgery in pediatrics?

Less than 5 years ago

5 years ago

More than 5 years ago

More than 10 years ago

Frequency (per week/per month/per year)?

1 per week

2 to 4 per week

1 per month

6 to 10 per month

10 to 20 per year

Other (please specify):

Most frequent age group:

3-5 years

5-8 years

9-11 years

12-15 years

Other (please specify):

What were the primary symptoms?

Severe headache

Intracranial hypertension (HTIC)

Seizures

Nausea and vomiting

Drowsiness/Fatigue

Personality, mood, or behavior changes

Difficulty speaking or understanding words

Difficulty swallowing/eating

Difficulty walking/weakness on one side of the body

Balance issues

Vision/hearing problems

Other (please specify):

What are possible contraindications for pediatric awake brain surgery?

Age

Tumor type

Tumor extent

Brain maturation level

Children with special needs

Other (please specify):

What is your opinion on the role of the speech-language pathologist in pediatric awake brain surgery?

Unimportant

Optional

Essential

Which professionals are essential during awake pediatric brain surgery?

Neuropsychologist

Neurologist

Psychologist

Psychomotor Therapist

Physical Therapist

Occupational Therapist

Speech-Language Pathologist

Other (please specify):

→ At which stage?

Preoperative Phase

Intraoperative Phase

Postoperative Phase

In your experience, what is the best age for pediatric awake brain surgery?

3 years

7 years

9 years

12 years

Other (please specify):

Is awake surgery the same for a child in the developmental learning stage compared to a child with academic skills?

Yes

No

What types of lesions/tumors indicate awake brain surgery in pediatrics?

Low-grade glioma

High-grade glioma

Arteriovenous malformation

Epilepsy

Metastasis

Dysplasia

Cavernoma

Other (please specify):

How long does it take to stimulate a brain area?

Less than 1 second

1 second

More than 1 second (please specify):

What is the possible stimulation frequency for each area?

Once

Twice, non-consecutive

Twice, consecutive

Three times, consecutive

Three times, non-consecutive

Other (please specify):

What are the risks if stimulation duration exceeds the maximum?

Seizures

False positives

False negatives

Transient brain injury

Irreversible brain injury

Transient function loss

Permanent function loss

Coma

Other (please specify):

Are there any risks even after completing mapping?

Yes

No

Was a speech-language pathologist present during your first awake surgery?

Yes

No

→ If no, how long did you perform awake surgery without a speech-language pathologist?

Less than 6 months

1 year

More than 1 year

For multilingual children, what specific considerations are taken during surgery?

What is the best position for intraoperative evaluation?

Supine

Lateral

Other (please specify):

Is psychological support provided immediately after surgery?

Yes

No

Is the patient’s quality of life taken into account?

Yes

No

What post-surgical sequelae may occur?

None

Tumor recurrence

Seizures

Neurolinguistic disorders (aphasia, dysarthria)

Motor impairments

Sensory deficits

Sensory disturbances

Other (please specify):

In postoperative care, is speech or neuropsychological rehabilitation:

Systematic

Frequent

Recommended

Is there a difference in post-surgical experience between adults and children, or does it depend on the individual?

Yes

No

Please explain how this manifests:

How is the role of parents of a child considered before, during, and after awake surgery?

Do you consider therapeutic education for the patient and family essential?

Yes

No

III. Remarks and Suggestions:

Survey for speech-language pathologists

We are conducting research on " Speech-Language Pathology Intervention in Pediatric Awake Brain Surgery: A Global Perspective." With limited professionals involved in pediatric awake brain surgery in Morocco, we are extending this survey internationally. The goal is to understand the practices of professionals in this specialized field.

Your responses will be treated confidentially and anonymously. Thank you for your time and participation.

I. Demographic and Professional Information:

Gender:

Female

Male

Country of Practice:

Morocco

France

Spain

United States

Lebanon

Tunisia

Algeria

Canada

Belgium

Italy

United Arab Emirates

Other (please specify):

Years of Professional Experience:

<5 years

=5 years

>5 years

Sector of Work:

Public

Private

Primary Education Institution:

Public

Private

In Morocco

Abroad

Workplace/Facility: (Please specify)

II. Speech-Language Pathology Intervention in Pediatric Awake Brain Surgery:

Have you received training in awake brain surgery and/or brain mapping?

Yes

No

If yes:

Training Type:

Initial Training

Continuing Education

Personal Initiative

Training Location:

France

Spain

USA

Italy

Mexico

Morocco

Lebanon

Algeria

Tunisia

Other (please specify):

Trainer’s Name (if available):

Training Duration:

1 month

3 months

6 months

1 year

2 years

Other (please specify):

If no:

Would you be interested in receiving this training?

Yes

No

Importance of Training in This Area:

Essential

Beneficial

Should be mandatory

Years of Experience in Pediatric Awake Brain Surgery:

<5 years

=5 years

>5 years

>10 years

According to your experience, the ideal team for awake brain surgery should include:

Neurosurgeon

Anesthesiologist

Speech-Language Pathologist

Psychomotor Therapist

Physiotherapist

Psychologist

Neuropsychologist

Other (please specify):

Are you currently part of a pediatric awake brain surgery team?

Yes

No

If no, why?

Lack of training in this area

Inadequate equipment/evaluation tools

Specific challenges of the child’s brain

High risk of complications

Other (please specify):

If yes:

Institution:

Years in Team:

<5 years

=5 years

>5 years

>10 years

Number of pediatric patients assessed to date:

Frequency of Assessments:

1 per week

2-4 per week

1 per month

6-10 per month

10-20 per year

Other (please specify):

Most Common Age Group:

3-5 years

5-8 years

9-11 years

12-15 years

Other (please specify):

III. Preoperative Indicators and Evaluation:

Common Symptoms Indicating Need for Surgery:

Intense headaches

Intracranial hypertension (HTIC)

Seizures

Nausea and vomiting

Drowsiness/fatigue

Changes in personality, mood, and behavior

Difficulty in speech or comprehension

Difficulty swallowing/eating

Difficulty walking/weakness on one side of the body

Balance issues

Vision or hearing problems

Other (please specify):

Contraindications for Pediatric Awake Brain Surgery:

Age

Tumor type

Tumor extent

Brain maturation

Children with special needs

Other (please specify):

Speech-Language Pathologist's Role in Pediatric Awake Brain Surgery:

Not important

Optional

Essential

Essential Professionals in Pediatric Awake Brain Surgery:

Neuropsychologist

Neurologist

Psychologist

Psychomotor Therapist

Physiotherapist

Occupational Therapist

Other (please specify):

Preferred Stages for Professional Involvement: (Please indicate the stages for each professional)

Professional

Preoperative

Peroperative

Postoperative

Neuropsychologist

Neurologist

Psychologist

Physiotherapist

Psychomotor Therapist

Occupational Therapist

Experiences in Pediatric Awake Brain Surgery

In your experience, what is the most appropriate average age for performing pediatric awake brain surgery?

3 years

7 years

9 years

12 years

Other (please specify):

How long does the stimulation of a brain area last?

<1 second

=1 second

>1 second (please specify):

How often can each brain area be stimulated?

Once

Twice, non-consecutively

Twice, consecutively

Three times, consecutively

Three times, non-consecutively

Other (please specify):

What is the probable risk if you exceed the maximum stimulation duration?

Seizures

False positives

False negatives

Temporary brain injury

Irreversible brain injury

Temporary loss of function

Permanent loss of function

Coma

Other (please specify):

Is there a risk even after mapping is completed?

Yes

No

Is awake surgery for a developing child the same as for a child with established school-based skills?

Yes

No

Does the child receive psychological support before surgery?

Yes

No

What tools are used for psychological preparation?

Explanation

Diagrams

Video

Therapeutic education program

Meeting with children who have had the surgery

Other (please specify):

For school-aged children, do you assess written language?

Yes

No

If no, why not?

Patient positioning

Time constraints

Lack of suitable tools

Patient must remain still

Other (please specify):

If yes, what aspects do you evaluate?

Reading

Reading comprehension

Writing (dictation and copying)

Graphic skills

Spelling

Other (please specify):

At what stage?

Preoperative

Perioperative

Postoperative

III. Evaluation of Neurocognitive Functions

What other neurocognitive functions should be evaluated?

Attention

Memory

Executive function

Visuospatial function

Visuotemporal function

Logical-mathematical reasoning

Praxis

Social cognition

Fine motor skills

Gnosis

Other (please specify):

At what stage are these functions evaluated?

Preoperative

Perioperative

Postoperative

What aspects of oral language do you evaluate?

Identification

Naming

Repetition

Spontaneous speech

Oral comprehension

Automatic language

Verbal fluency

Storytelling

Other (please specify):

At what stage?

Preoperative

Perioperative

Postoperative

IV. Behavioral and Mood Assessments

Are behavioral or mood evaluations conducted?

Yes

No

If yes, are these conducted perioperatively?

Yes

No

V. Practical Aspects and Challenges

Is it sometimes impossible to complete all planned tests?

Regularly

Rarely

Never

If yes, what are the reasons?

Anxiety and fear

Fatigue

Surgical complications

Pain

Effects of sedation and anesthesia

Insufficient time

Deep electrical stimulation

Other (please specify):

In which language are the tests conducted?

Native language

Acquired language(s)

Both

What is the maximum duration for test completion/mapping?

What type of equipment do you use?

No specific equipment

Self-designed/adapted equipment

Tools from assessment batteries

Other (please specify):

What format is used for presenting materials?

Paper

Digital

Are preoperative assessments the same as those in perioperative and postoperative phases?

Yes

No

If yes, when are they conducted preoperatively?

1 day before surgery

2 days before surgery

1 week before surgery

2 weeks before surgery

Other (please specify):

And postoperatively? - 1 day after surgery - 2 days after surgery - 1 week after surgery - 2 weeks after surgery - Other (please specify):

VI. Assessment Tools and Language Skills

Which materials do you use?

DO 80 (Oral Naming of Images)

DRA (Rapid Naming for Adults and Children)

BETL (Lexical Troubles Evaluation Battery)

BDAE (Boston Diagnostic Aphasia Examination)

Vol du PC (Reading Function Assessment for ages 11-18)

NEPSY (Neuropsychological Battery for Children and Adolescents)

ALOUETTE (Reading and Dyslexia Analysis Test)

EDINBURGH (Manual Laterality Questionnaire)

L2MA (Oral and Written Language, Memory, Attention)

TLOCC (Complex Oral Language Test for Adolescents)

PELEA (Elaborate Language Assessment Protocol for Adolescents)

BALE (Analytical Battery for Written Language)

DEN 84 (Naming and Verbal Fluency Test)

ELO (Oral Language Assessment)

CHRONODICTÉ (Transcription Abilities Assessment in Various Domains of Orthography, Ages 7 to Adult)

Other (please specify):

Do you evaluate spontaneous language through:

Conversational language

Storytelling

Image description

Other (please specify):

Do you evaluate naming through:

Images

Numbers

Actions

Other (please specify):

Do you evaluate repetition through:

Simple words

Complex words

Logatomes

Numbers

Simple sentences

Complex sentences

Other (please specify):

Do you evaluate automatic language through:

Counting

Days of the week

Months of the year

Seasons

Letters

Fable or song

Other (please specify):

Do you assess reading through:

Alphabets

Words

Numbers

Texts

Irregular/regular words

Logatomes

Other (please specify):

Do you test writing during awake surgery?

Yes

No

If yes, in which position?

Supine

Lateral decubitus

Other (please specify):

With which format of tools?

Digital/touchscreen

Paper and pencil

Why is it relevant to evaluate writing/reading skills in children?

For a multilingual child, what specific factors do you consider during pre-, peri-, and postoperative evaluations?

What sequelae may occur after surgery?

None

Tumor recurrence

Seizures

Neurolinguistic disorders (aphasia, dysarthria)

Motor impairments

Sensory impairments

Sensory-motor impairments

Other (please specify):

Postoperative speech therapy is:

Systematic

Frequent

Recommended

Is there a difference in the experience after awake brain surgery between adults and children, or does it vary by patient?

Yes

No

If yes, please describe:

How is parental involvement accounted for before, during, and after the awake brain surgery?

Do you believe therapeutic education for the patient and family is essential?

Yes

No

VIII. Remarks and Suggestion :

Questionnaire for Other Professionals (Anesthesiologists - Neuropsychologists - Psychologists - Physiotherapists - Psychomotor Therapists - Occupational Therapists - Nurses…)

We are currently conducting research on " Speech-Language Pathology Intervention in Pediatric Awake Brain Surgery: A Global Perspective." Given the limited number of professionals involved in awake pediatric surgery in Morocco, our survey also extends internationally. The goal is to better understand the practices of speech-language pathologists and other professionals involved in this specific field. To this end, we are sending you this questionnaire, which is intended for professionals (speech-language pathologists, neurosurgeons, neuropsychologists, anesthesiologists, physiotherapists, psychomotor therapists, psychologists) involved in awake pediatric brain surgery. Your responses will be invaluable to enrich our research. We assure you that your responses will be treated confidentially and anonymously. We thank you for the time and attention you dedicate to this survey.

Demographic and Professional Information:

What is your profession?

Neuropsychologist

Psychologist

Psychomotor Therapist

Neurologist

Anesthesiologist

Physiotherapist

Occupational Therapist

Nurse

Other (please specify):

In which country do you practice?

Morocco

France

Spain

United States of America

Lebanon

Tunisia

Algeria

Canada

Belgium

Italy

United Arab Emirates

Other (please specify):

How long have you been practicing?

< 5 years

= 5 years

·        5 years

What is your sector of work?

Public

Private

Where was your basic training?

Public

Private

In Morocco

Abroad

In which type of institution do you practice?

I. Speech-Language Therapy Intervention in Awake Pediatric Brain Surgery:

Have you ever received training in awake surgery and/or brain mapping?

Yes

No

→ If yes:

In what context?

Initial training

Continuing education

Personal initiative

Please specify where you received this training:

France

Spain

USA

Italy

Mexico

Morocco

Lebanon

Algeria

Tunisia

Other (please specify):

If possible, specify who provided the training:

What was the duration of this training?

1 month

3 months

6 months

1 year

2 years

Other (please specify):

→ If no, would you be willing to receive training in this area?

Yes

No

Do you believe this training is:

Fundamental

Relative

Should be mandatory

How many years have you been part of the awake brain surgery team?

< 5 years

= 5 years

>5 years

>10 years

In your experience, the ideal team for awake surgery should include:

A neurosurgeon

An anesthesiologist

A speech-language pathologist

A psychomotor therapist

A physiotherapist

A psychologist

A neuropsychologist

Other (please specify):

Have you ever participated in awake pediatric brain surgery?

Yes

No

→ If no, why?

No training in the area

Lack of adequate equipment

Specificities of the child’s brain

Major risks and sequelae

Other (please specify):

→ If yes:

Please specify the institution where you participated:

How long have you been participating?

<5 years

=5 years

>5 years

>10 years

With how many children have you been involved? __________

Frequency (per week / per month / per year)?

1 / week

2 to 4 / week

1 / month

6 to 10 / month

10 to 20 / year

Other (please specify):

What is the most common age group?

3-5 years

5-8 years

9-11 years

12-15 years

Other (please specify):

What do you think of the role of the speech-language pathologist in awake pediatric brain surgery?

Unimportant

Optional

Essential

Which professionals are essential during awake pediatric surgery?

Neuropsychologist

Neurologist

Psychologist

Psychomotor Therapist

Physiotherapist

Occupational Therapist

Speech-Language Pathologist

Other (please specify):

→ And at which stage?

Preoperative Phase

Intraoperative Phase

Postoperative Phase

Is awake surgery the same for a child in the development and learning phase as for a child with academic acquisitions?

Yes

No

Is the presence of a speech-language pathologist alone sufficient for conducting pre-, intra-, and postoperative assessments?

Yes

No

How is the patient prepared for surgery?

Through explanations

Using diagrams

Using videos

Through assessments

Other (please specify):

Which professionals are involved in this preparation?

Neurosurgeon

Neuropsychologist

Anesthesiologist

Speech-Language Pathologist

Psychiatrist

Psychologist

Psychomotor Therapist

Physiotherapist

Occupational Therapist

Nurses and healthcare assistants

Other (please specify):

What are their respective roles? Roles

Professionals

Roles

Neurosurgeon

Neuropsychologist

Anesthesiologist Psychologist Speech-Language Pathologist Psychomotor Therapist Physiotherapist Occupational Therapist Nurses and healthcare assistants Psychiatrist/Child Psychiatrist

Is the intraoperative assessment assisted by:

A speech-language pathologist

A neuropsychologist

A physiotherapist

A psychologist

A psychomotor therapist

Other (please specify):

Is the postoperative assessment assisted by:

A speech-language pathologist

A neuropsychologist

A physiotherapist

A psychologist

A psychomotor therapist

Other (please specify):

For a complete assessment during awake pediatric surgery, can the speech-language pathologist, neuropsychologist, physiotherapist, or others conduct a joint assessment at the same time?

Yes

No

If the assessment is conducted by the speech-language pathologist, the roles of other professionals are:

Observers

Assistants

Interveners

Other (please specify):

What cognitive tasks can be highlighted?

Attention

Memory

Executive Function

Visuospatial Function

Visuotemporal Function

Logical-mathematical reasoning

Praxias

Social Cognition

Fine Motor Skills

Gnosis

Oral/Written Language

Other (please specify):

Can other evaluations of behavior or mood be performed?

Yes

No

What is your role in awake pediatric brain surgery?

In the preoperative phase:

In the intraoperative phase:

In the postoperative phase:

Which tests do you use?

Preparation tools:

Evaluation tools:

Follow-up tools:

What is the best position for intraoperative evaluation?

Supine

Lateral

Other (please specify):

Is the intraoperative evaluation exactly the same as the pre- and postoperative evaluations?

Yes

No

Is psychological follow-up established immediately after the intervention?

Yes

No

Is the patient’s quality of life taken into account?

Yes

No

What sequelae can be expected after the surgery?

None

Tumor recurrence

Seizures

Neurolinguistic disorders (aphasia, dysarthria)

Motor disorders

Sensory disorders

Sensory disturbances

Other (please specify):

In the postoperative phase, is speech-language or neuropsychological rehabilitation:

Systematic

Frequent

Advised

Is there a difference in the experience (after awake surgery) between adults and children, or does it depend on each patient?

Yes

No

Can you specify how this manifests?

In your opinion, how is the role of parents considered before, during, and after awake surgery?

Do you think therapeutic education for the patient and their family is essential?

Yes

No

II. Comments and Suggestions:
